# Suppression of Hypertrophy During *in vitro* Chondrogenesis of Cocultures of Human Mesenchymal Stem Cells and Nasal Chondrocytes Correlates With Lack of *in vivo* Calcification and Vascular Invasion

**DOI:** 10.3389/fbioe.2020.572356

**Published:** 2021-01-05

**Authors:** Matthew Anderson-Baron, Yan Liang, Melanie Kunze, Aillette Mulet-Sierra, Martin Osswald, Khalid Ansari, Hadi Seikaly, Adetola B. Adesida

**Affiliations:** ^1^Division of Orthopaedic Surgery, Department of Surgery, Faculty of Medicine and Dentistry, Laboratory of Stem Cell Biology and Orthopaedic Tissue Engineering, University of Alberta, Edmonton, AB, Canada; ^2^Division of Surgical Research, Department of Surgery, Faculty of Medicine and Dentistry, Laboratory of Stem Cell Biology and Orthopaedic Tissue Engineering, University of Alberta, Edmonton, AB, Canada; ^3^Division of Otolaryngology-Head and Neck Surgery, Department of Surgery, Faculty of Medicine and Dentistry, University of Alberta Hospital, Edmonton, AB, Canada; ^4^Institute for Reconstructive Sciences in Medicine, Misericordia Community Hospital, Edmonton, AB, Canada

**Keywords:** cartilage, cocultures, human mesenchymal stem cells, hypertrophic chondrogenesis, human nasal chondrocytes, xenograft, *in vivo* calcification, vascular invasion

## Abstract

**Objective:**

Human nasal septal chondrocytes (NC) are a promising minimally invasive derivable chondrogenic cell source for cartilage repair. However, the quality of NC-derived cartilage is variable between donors. Coculture of NC with mesenchymal stem cells (MSCs) mitigates the variability but with undesirable markers of chondrocyte hypertrophy, such as type X collagen, and the formation of unstable calcifying cartilage at ectopic sites. In contrast, monoculture NC forms non-calcifying stable cartilage. Formation of a stable NC-MSC coculture cartilage is crucial for clinical application. The aim of this study was to explore the utility of parathyroid hormone-related peptide (PTHrP) hormone to suppress chondrocyte hypertrophy in NC-MSC cocultures and form stable non-calcifying cartilage at ectopic sites.

**Methods:**

Human NC and bone marrow MSCs, and cocultures of NC and MSC (1:3 ratio) were aggregated in pellet form and subjected to *in vitro* chondrogenesis for 3 weeks in chondrogenic medium in the presence and absence of PTHrP. Following *in vitro* chondrogenesis, the resulting pellets were implanted in immunodeficient athymic nude mice for 3 weeks.

**Results:**

Coculture of NC and MSC resulted in synergistic cartilage matrix production. PTHrP suppressed the expression of hypertrophy marker, type X collagen (*COL10A1*), in a dose-dependent fashion without affecting the synergism in cartilage matrix synthesis, and *in vivo* calcification was eradicated with PTHrP. In contrast, cocultured control (CC) pellets without PTHrP treatment expressed *COL10A1*, calcified, and became vascularized *in vivo*.

## Introduction

Advances in the field of tissue engineering have made tissue-engineered cartilage a promising option for the treatment or replacement of damaged and diseased cartilage tissue. However, considerable challenges remain, including the acquisition of appropriate autologous sources of cells and the generation of stable engineered cartilage once implanted *in vivo*. Nasal chondrocytes (NC) have become an attractive source of chondrocytes for generating tissue engineered cartilage (Candrian et al., 2008; Acharya et al., 2012; Scotti et al., 2012; Fulco et al., 2014). Nasal septal cartilage is relatively easy to isolate from the septum and the NC derived from septal cartilage have been shown to proliferate rapidly in culture while maintaining their chondrogenic lineage (Fulco et al., 2014; Mumme et al., 2016). The use of fibroblast growth factor-2 (FGF2) and transforming growth factor β-1 (TGFβ-1) supplemented growth media during NC expansion has been shown to facilitate chondrogenic redifferentiation and the expression of functional cartilage extracellular matrix (ECM) in NC (Tay et al., 2004; Pelttari et al., 2014; Andrews et al., 2017). NC have been shown to produce stable cartilage constructs *in vivo* and have been used to repair articular cartilage of the knee and reconstruct nasal cartilage in skin cancer patients (Fulco et al., 2014; Mumme et al., 2016); however, engineered cartilage from NC suffers from large donor-to-donor variability (Fulco et al., 2014; Andrews et al., 2017).

Coculture is a method of culturing two or more distinct cell types in the same environment, which has been shown to have beneficial effects from direct cell-to-cell contacts in cartilage tissue engineering (Acharya et al., 2012; Paschos et al., 2015). It has been demonstrated that the coculture of NC with bone marrow-derived mesenchymal stem cells (BM-MSCs) produces a synergistic effect, whereby sulfated glycosaminoglycan (GAG) ECM production is increased (Wu et al., 2011; Acharya et al., 2012). Cooke et al. (2011) showed that the three-dimensional (3D) coculture of BM-MSC with juvenile chondrocytes showed increased GAG production and increased expression of chondrogenic genes, such as *SOX9*, *aggrecan* (*ACAN*), and *type II collagen* (*COL2A1*). This synergism is termed chondro-induction whereby the GAG production observed is greater than the sum of the GAG production expected from each cell type alone (Acharya et al., 2012). Furthermore, coculture of NC and BM-MSC has been shown as an effective strategy to mitigate the donor-to-donor variability in NC derived engineered cartilage. However, it has been shown that the engineered cartilage from cocultured BM-MSC and NC are prone to undergo calcification when implanted in nude mice in similitude to implanted engineered cartilage microtissues from chondrogenically-induced BM-MSC (Pelttari et al., 2006; Andrews et al., 2017). In contrast, the engineered cartilage from NC alone did not undergo *in vivo* calcification (Acharya et al., 2012). Pelttari et al. (2006) had demonstrated that the *in vivo* calcification of BM-MSC-derived cartilage microtissues correlated with their expression of molecular markers of chondrocyte hypertrophy such as type X collagen (*COL10A1*) and matrix metalloproteinase 13 (*MMP13*).

Parathyroid hormone-related peptide (PTHrP) has been shown to suppress the hypertrophic differentiation of BM-MSC (Kafienah et al., 2007). Kafienah et al. (2007) demonstrated that treatment of BM-MSC with 1 or 10 μM PTHrP significantly reduced *COL10A1* expression and alkaline phosphatase activity. In addition, it has been shown that BM-MSC cultured in media conditioned by human articular chondrocytes showed decreased expression of hypertrophic markers, such as *COL10A1* and alkaline phosphatase, which was attributed to chondrocyte-derived PTHrP released into the media (Fischer et al., 2010). It has been suggested that PTHrP plays a role in suppressing late chondrogenesis toward hypertrophic differentiation (Weiss et al., 2010). The PTHrP/Indian hedgehog (IHH) pathway operates a feedback loop that is involved in the maintenance of the chondrocyte phenotype in the growth plate of skeletal tissue and helps maintain healthy articular cartilage tissue (Kronenberg, 2003). PTHrP exerts its effects by binding to the PTHrP receptor (PTHR), a G-protein coupled receptor that activates the adenylyl cyclase/protein kinase A (PKA) downstream signaling pathway (Mannstadt et al., 1999). Activated PKA exerts downstream effects, deactivating the transcription of hypertrophic genes and maintaining chondrocytes in a proliferative state (Caron et al., 2015; Ripmeester et al., 2018). It is also thought that PTHrP activates the transcription of BAPX1/NKX3.2 (Provot et al., 2006), which is a potent hypertrophic switch in chondrocytes (Caron et al., 2015).

Because PTHrP has been demonstrated to suppress hypertrophic differentiation of chondrocytes, we sought to examine the use of PTHrP to prevent the hypertrophy and subsequence calcification of cocultured NC and BM-MSC-derived tissue engineered cartilage. Tissue engineered microtissue cartilage constructs were formed from cell aggregate pellet cultures of human NC, BM-MSC, and cocultures of NC and BM-MSC (Anderson-Baron et al., 2020). Cocultured microtissue pellets were treated with increasing concentrations of recombinant human PTHrP. PTHrP treatment appeared to decrease the expression of hypertrophic marker-*COL10A1*. In addition, upon subcutaneous implantation of the microtissue pellets into immunodeficient nude mice, pre-treatment of cocultured pellets with PTHrP resulted in a suppression of vascularization and calcification. Our results demonstrate the utility of PTHrP treatment in suppressing hypertrophic differentiation and calcification of cocultured NC and BM-MSC engineered cartilage.

## Materials and Methods

### Cell Harvest

All human tissues specimens were collected from surgically discarded material after approval and waiver of informed consent of the University of Alberta’s Health Research Ethics Board (Study Protocol ID: Pro00018778). Nasal septal cartilage was collected from nine donors (three females, six males; age range: 23–52 years of age; average age: 34.67 years) at the Leduc Community Hospital, Alberta, Canada. Human bone marrow aspirates were collected from the iliac crest of eight donors (five females and three males; age range: 26–59 years of age; average age: 42.25 years) undergoing routine orthopedic surgery at the University of Alberta Hospital and the Misericordia Community Hospital in Edmonton, Alberta, Canada. All donor information can be found in Supplementary Tables S1, S2.

### Cell Isolation and Culture

Nasal septal cartilage was enzymatically digested in 0.15% (w/v) type II collagenase (300 units/mg; Worthington Biochemical Corporation) in Dulbecco’s Modified Eagle Medium (DMEM; Sigma) supplemented with heat inactivated fetal bovine serum (FBS; 5% v/v; Gibco), 100 U/mL penicillin and streptomycin with L-glutamine (2 mM; Thermo Fisher), and HEPES (0.1 M; Thermo Fisher). Samples were incubated for 22 h at 37°C in a shaking incubator at 250 r/min. Digested tissue was passed through a 70-μm nylon strainer and undigested particulate tissue was removed. NC released from the tissue digests were washed three times in sterile phosphate buffered saline (PBS; Sigma), resuspended in standard media, and plated in tissue culture flasks in a humidified incubator under normal atmospheric conditions (normoxic; 21% O_2_, 5% CO_2_, 95% humidity). After 24 h, adherent cells were removed by trypsinization and the cell populations were counted using a hemocytometer and Trypan Blue. After isolation, NC were plated at 10,000 cells/cm^2^ in tissue culture flasks. NC were expanded in normoxic conditions at 37°C in DMEM complete [supplementation with heat inactivated FBS (10% v/v; Gibco), 100 U/ml penicillin and streptomycin with L-glutamine (2 mM; Thermo Fisher), and HEPES (0.1 M; Thermo Fisher)], supplemented with 1 ng/mL TGF-β1 (Prospec), and 5 ng/mL FGF-2 (Prospec). Expansion culture media was changed twice a week. NC were used for experiments at the end of passage one (P1), with an average cumulative population doubling of 4.12 (range: 1.62–7.66).

Bone marrow mononucleated cells (BM-MNCs) were isolated from bone marrow aspirates using Histopague-1077 (Sigma) according to the manufacturer’s instructions. In brief, donor bone marrow aspirate was diluted 1:1 in PBS. The diluted sample was layered over top of Histopaque-1077 at a 2:1 ratio (sample: Histopaque-1077). Samples were centrifuged at 1200 r/min for 30 min at 25°C. BM-MNCs were isolated from the interface of the plasma-Histopaque-1077 layers and diluted 1:3 in α Minimum Eagle’s Medium (αMEM; Gibco) complete [i.e., supplementation with heat inactivated FBS (10% v/v; Gibco), 100 U/mL penicillin and streptomycin with L-glutamine (2 mM; Thermo Fisher), and HEPES (0.1 M; Thermo Fisher)]. Diluted samples were centrifuged at 1500 r/min for 10 min at 25°C. The resulting supernatant was aspirated, and cell pellets were resuspended in 2–5 mL of αMEM complete (volume dictated by the size of the pellet). Samples were again centrifuged at 1200 r/min for 5 min at 25°C. BM-MNC numbers were determined using Crystal Violet solution (Sigma; 1:50 dilution in PBS) and a hemocytometer. Cells were then plated at 10,000 cells/cm^2^ in αMEM complete with 5 ng/mL FGF-2 at 37°C and 5% CO_2_. Media changes were performed twice a week. A colony-forming unit fibroblastic (Cfu-f) assay was performed in parallel to determine the proportion of BM-MNC within the marrow aspirates forming plastic adherent BM-MSC clones and subsequent population doubling of the inherent BM-MSCs (Solchaga et al., 2010). Briefly, Cfu-f was performed by plating 100,000 BM-MNC per100 mm petri dishes (in triplicates) in αMEM complete with 5 ng/mL FGF-2 at 37°C and 5% CO_2_ for 2 weeks. BM-MSCs were used for experiments at the end of passage 2 (P2) with an average cumulative population doubling of 14.84 (range: 13.46–18.98).

### Cell Aggregate Pellet Culture

Cell pellets were formed by centrifugation at 433 g for 5 min, with either 2.5 × 10^5^ or 5 × 10^5^ cells per pellet. Three different groups based on cell type were formed: monoculture NC, monoculture BM-MSC, and cocultured pellets with NC and BM-MSC combined at a ratio of 1:3 NC:BM-MSC. The pellets were cultured in a defined serum-free chondrogenic media, composed of DMEM supplemented with 100 U/mL penicillin and streptomycin, 2 mM L-glutamine, 100 mM HEPES, insulin-transferrin-selenium (ITS+1), 0.1 μM dexamethasone, 0.1 mM ascorbic acid 2-phosphate, 0.1 mM L-proline, and 10 ng/mL TGF-β1. Pellet cultures were maintained for 3 weeks and culture media was changed twice a week. Cocultured pellets were untreated (control) or treated with 2, 20, or 200 ng/mL of PTHrP (residues 1-34; BACHEM, 4017147.500). PTHrP treatment was repeated at each media change.

### Gross Morphology of Pellets

The gross appearance of pellets was captured after 3 weeks of *in vitro* culture in chondrogenic medium as stated above. Gross images were capture using Carl Zeiss Stemi 2000 stereomicroscope fitted with an AxioCam ERc5s digital camera.

### Sulfated Glycosaminoglycan and DNA Quantification

After 3 weeks of culture in chondrogenic media, pellets were rinsed in PBS and frozen at −80°C. Pellets were thawed and digested in 1 mg/mL Proteinase K (Sigma) overnight at 56°C. In order to quantify the GAG content, a 1,9-dimethylmethylene blue (DMMB; Sigma) assay was performed, with chondroitin sulfate (Sigma) used as the standard. Colorimetric readings were measured at 530 nm using a V-max kinetic microplate reader (Molecular Devices). DNA content from Proteinase K digests was measured using the CyQUANT Cell Proliferation Assay Kit (Thermo Fisher). Measurements were taken according to the manufacturer’s instruction. The supplied λ-bacteriophage DNA was used as a standard. Fluorescence emission was measured at 530 nm (excitation 450 nm) on a CytoFluor II fluorescence multi-well plate reader (PerSeptive Biosystems).

An interaction index was calculated, as per Acharya et al. (2012). In brief, the Interaction Index = GAG_*Measured*_/ GAG_*Expected*_, where GAG_*Expected*_ = (GAG_*NC*_^∗^ 0.25) + (GAG_*BM–MSC*_^∗^ 0.75). GAG_*Expected*_ was calculated as the ratio of NC:BM-MSC in cocultured pellets, whereby 25% were NC and 75% were BM-MSCs. Cocultures resulting in interaction index > 1 were termed as Responders and those with interaction index < 1 as Non-Responders.

### Histology

Pellets were fixed in 10% (v/v) formalin (Anachemia) overnight at 4°C, washed in PBS, processed, and embedded in paraffin wax. Embedded pellets were cut at 5-μm thickness using a Leica RM2125 RTS rotary microtome (Leica Biosystems). Sections were deparaffinized using Xylene Substitute (VWR International) and stained with 0.1% (w/v) Safranin-O (Sigma). Sections were then counter-stained with 1% (w/v) Fast Green FCF (Sigma) and mounted in Richard-Allan Scientific Mounting Medium (Thermo Fisher). The resulting stained sections were imaged on a Nikon Eclipse Ti-S microscope coupled to a DS-U3/Fi2 Color CCD camera using 10x and 20x objective lenses.

For calcium staining, sections were deparaffinized, as described above. Sections were stained with Alizarin Red S (Sigma–Aldrich; A5533) solution [2% (w/v), pH 4.2] for 5 min. Sections were then dehydrated in acetone and acetone: xylene 1:1 solution. Finally, sections were cleared in UltraClear xylene Substitute (VWR International), mounted, and imaged as described above.

### Immunofluorescence

Pellets were fixed, embedded, and sectioned, as described above. Sections were deparaffinized as described above and progressively rehydrated in ethanol [100% (anhydrous), 95% v/v, 70% v/v, and 35% v/v]. Following complete rehydration in water, antigen retrieval was performed with 1 mg/mL protease XXV (Thermo Fisher) for 30 min at room temperature. Sections were washed in PBS and treated with 1 mg/mL Hyaluronidase (Sigma) for 30 min at 37°C. Sections were blocked in 5% (w/v) bovine serum albumin (BSA; Cell Signaling Technology) for 30 min at room temperature. Type I collagen (rabbit; CL50111AP; Cedar Lane) and type II collagen (mouse; II-II6B3; Developmental Studies Hybridoma Bank) primary antibodies [1:100 dilution in 1% (w/v) BSA] were incubated with sections overnight at 4°C. Sections were washed thoroughly in PBS and incubated for 45 min at room temperature with goat anti-rabbit IgG Alexa Fluor 594 (ab150080; Thermo Fisher) and goat anti-mouse IgM Alexa Fluor 488 (ab150121; Thermo Fisher) secondary antibodies [dilution 1:200 in 1% (w/v) BSA]. Sections were washed thoroughly in PBS and incubated in 4’,6-diamidino-2-phenylindole (DAPI) for 5 min at room temperature. Sections were mounted with 1:1 Glycerol: PBS and imaged on a Nikon Eclipse Ti-S microscope coupled to a DS-U3/Fi2 Color CCD camera using 10x and 20x objective lenses.

For type X collagen (COL X) immunofluorescence (IF), pellets were deparaffinized, rehydrated, and treated with protease XXV and Hyaluronidase, as described above. Following the Hyaluronidase treatment, sections were treated with 0.2% (v/v) Triton-X for 10 min at room temperature. Sections were blocked in 5% (w/v) BSA (Cell Signaling Technology) for 30 min at room temperature. Type X collagen (rabbit; Abcam, ab58632) primary antibody [1:100 dilution in 1% (w/v) BSA] was incubated with sections overnight at 4°C. Sections were washed thoroughly in PBS and incubated with goat anti-rabbit IgG Alexa Fluor 594 (ab150080; Thermo Fisher) for 45 min at room temperature. Sections were washed, incubated with DAPI, mounted, and imaged, as described above.

For CD31 IF, pellets were deparaffinized, rehydrated as described above. Antigen retrieval was performed with citrate buffer [10 mM citric acid, 0.05% (v/v) Tween 20, pH 6.0] and incubated in an IHC Tek Epitope Retrieval Steamer set for 20 min. Sections were allowed to cool for 20 min to room temperature. Sections were washed thoroughly in PBS and blocked in 5% (w/v) BSA for 30 min. Sections were then incubated with biotinylated CD31 primary antibody (mouse) (R&D Systems; BAF3628) diluted at 1:200 in 1% (w/v) BSA overnight at 4°C. Sections were washed thoroughly in PBS and incubated with streptavidin-Alexa Fluor 488 conjugated secondary antibody (Life Technologies, S32354) at a 1:200 dilution in 1% (w/v) BSA for 45 min at room temperature. Sections were washed, incubated with DAPI, mounted, and imaged, as described above.

### Quantitative Real-Time Polymerase Chain Reaction (qRT-PCR)

Pellets were immersed in TRIzol reagent (Thermo Fisher) and frozen at −80°C. Samples were later thawed and cells were lyzed by grinding the pellets with a pestle. Total RNA was isolated by chloroform extraction. Complementary DNA (cDNA) was synthesized from 100 ng of total RNA using the GoScript Reverse Transcriptase kit (Promega) and 1 μg of oligo(dT) primers. Quantitative PCR was performed using Takyon DNA Polymerase and SYBR Green detection (Eurogentec) on a CFX Connect Real-Time PCR Detection System (Bio-Rad Laboratories). Primer sequences were designed using Primer Express 3.0.1 (Thermo Fisher). Primer sequences for *ACAN*, *ALPL*, *COL1A2*, *COL2A1*, *COL10A1*, *SOX9*, *MMP13*, *ID1*, *IHH*, *HMBS*, *B2M*, and *YWHAZ* are shown in Supplementary Table S3. Transcript levels for *ACAN*, *ALPL*, *COL1A2*, *COL2A1*, *COL10A1*, *SOX9*, *MMP13*, *ID1*, and *IHH* were normalized to the housekeeping genes *B2M*, *HMBS*, and *YWHAZ* using the delta delta CT method (2^–ΔΔ*CT*^) (Livak and Schmittgen, 2001; Schmittgen and Livak, 2008).

### Statistical Analysis

A total of nine independent experiments were performed with nine donors’ specimens for NC and eight donors’ specimens for BM-MSC. Unless stated specifically, numerical data distribution represents data from these donors each measured at least in independent duplicates or at most in triplicates and is presented as a bar graph of the mean ± standard deviation. Statistical analyses were performed using SPSS (version 26; IBM Canada Ltd., ON) and Microsoft Excel 2016. Data were tested for normality using the Shapiro–Wilk test, and Levene’s test was used to assess homogeneity of error variances. Statistical differences between the measured parameters of pellets formed from 250,000 (250K) and 500,000 (500K) cells were assessed by Student *t*-test. The relationship between measured parameters was determined by Pearson correlation coefficient based on confirmation of normality of data distribution. Statistically significant differences between multiple groups were assessed by one-way ANOVA and either adjusted by Tukey’s honest significance difference (HSD) or Games–Howell *post hoc* test depending on the outcome of the Levene’s test. Significance was considered when *p* < 0.05. The data were plotted in Excel in Microsoft Office 365 ProPlus.

### Animal Implantation

To evaluate *in vivo* calcification of pellets, the pellets were implanted into athymic nude CD-1 mice (*n* = 8, Charles River, Wilmington, MA, United States). Cell aggregate pellets were set up in triplicates with a total of 5 × 10^5^ cells as described above: NC, BM-MSC, coculture control, and coculture treated with 2 and 200 ng/mL PTHrP—see Supplementary Table S2. The pellets were cultured in chondrogenic media for 3 weeks, as previously described. After the 3 weeks, one pellet per the triplicate pellets was assayed for GAG, DNA, and interaction index. The remainder pellets were implanted subcutaneously in nude mice. All procedures were approved and undertaken under the auspices of and in accordance with the University of Alberta Animal Care and Use Committee. Four pellets were implanted subcutaneously (two cranial and two caudal) on the dorsal side of each mouse by a small incision. Each incision was closed by sutures and cyanoacrylate tissue adhesive. After 3 weeks of implantation, the pellets were retrieved with the mice under anesthesia. The mice were subsequently euthanized at experimental endpoint. Any excess mouse tissue was carefully removed from the pellets using a scalpel. The pellets were retrieved; their gross appearance was recorded, stored in PBS, and fixed in 10% (v/v) formalin, as previously described. The pellets were processed, embedded, and sectioned for histology, as previously described (Andrews et al., 2017).

## Results

### PTHrP Did Not Affect Chondrogenesis in Cocultured Pellets

**FIGURE 1:**
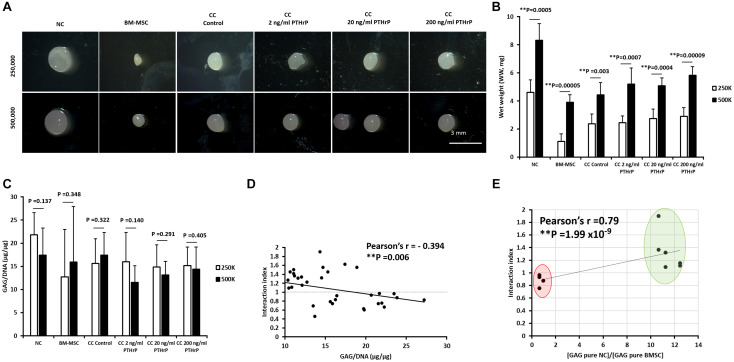
PTHrP did not affect chondrogenesis in cocultured pellets. Pellet cultures were either formed from 2.5 × 10^5^ or 5 × 10^5^ cells from nasal chondrocytes (NC), bone marrow-derived mesenchymal stem cells (BM-MSC), or cocultures consisting of NC and BM-MSC at a 1:3 ratio (CC). Pellets were cultured in chondrogenic media for 3 weeks without (control) or with PTHrP at 2, 20, or 200 ng/mL. After 3 weeks of culture, pellets were photographed, weighed, and analyzed. **(A)** Gross appearance images taken by brightfield microscopy. **(B)** Wet weights of pellets. **(C)** GAG per DNA content of pellets. **(D)** The Pearson bivariate correlation of interaction index (based on calculations from Acharya et al., 2012) with GAG per DNA contents. **(E)** The Pearson bivariate correlation of the interaction indices of control cocultured pellets with the ratio of GAG contents of monoculture (pure) of NC to GAG content of monoculture (pure) BM-MSC. Cocultured pellets with interaction indices < 1 and > 1 clustered in red and green oval shapes, respectively. Statistical significance was determined by Student *t*-test (six donors in total; four donors in duplicates at 250,000 cells per pellet and two donors in duplicates at 500,000 cells per pellet; *n* = 12; ^∗∗^*p* < 0.01).

**FIGURE 2:**
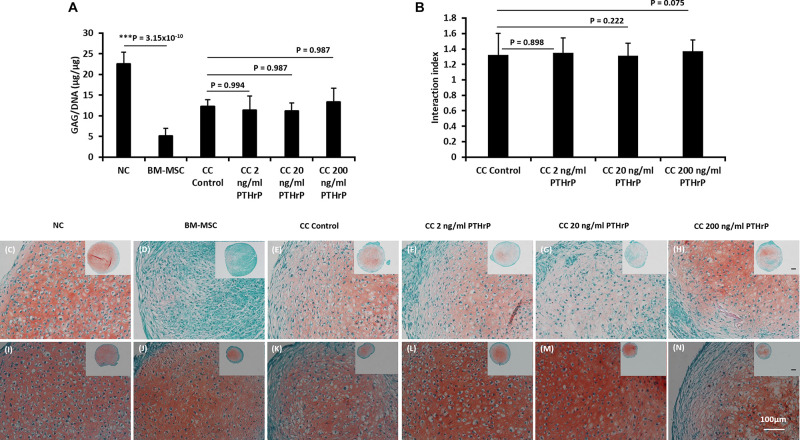
PTHrP does not affect Safranin-O staining in cocultured pellets with interaction index > 1 (i.e., Responders). After 3 weeks of culture in chondrogenic media, pellets were fixed, embedded, and sectioned for histology. **(A)** The GAG per DNA contents of the monoculture and cocultured pellets associated with interaction index > 1. **(B)** The interaction index of cocultured pellets. **(C–H)** Safranin O staining of pellets formed from 250,000 cells and **(I–N)** Safranin O staining of pellets formed from 500,000 cells. The main and inset images are at 200x and 100x optical magnification, respectively. Scale bars are 100 μm. Statistical significance was determined using one-way ANOVA with Tukey’s HSD *post hoc* test (three donors in total; two donors in duplicates at 250,000 cells per pellet and one donor in duplicates at 500,000 cells per pellet; *n* = 6; ***p < 0.001).

After 3 weeks of culture in chondrogenic media, pellets were harvested, the gross appearance of the pellets were captured on stereomicroscope (Figure 1A), the wet weight (WW) were recorded (Figure 1B) and *in vitro* chondrogenesis was first assessed by determining the GAG content relative to DNA content (Figure 1C). Regardless of whether the pellets were formed from a total of 250,000 or 500,000 cells, the pellets had a glassy cartilage-like appearance (Figure 1A). Monoculture BM-MSC pellets appeared the smallest (Figure 1A). All pellets formed from 500,000 cells had a higher WW than their 250,000 cells counterpart (Figure 1B). There was no significant difference in GAG/DNA contents between 250,000 and 500,000 cells derived pellets within experimental groups (Figure 2C).

In order to compare the relative levels of chondro-induction in cocultured pellets, an interaction index was quantified, as per Acharya et al. (2012). The interaction index provides an indication of the amount of GAG measured relative to the amount of GAG expected. Values > 1 indicate chondro-induction. The mean interaction index for some cocultured pellets, including cocultured control (CC; without PTHrP treatment) and PTHrP-treated, showed an interaction index value > 1, indicating chondro-induction. The cocultures with interaction indices > 1 were designated as Responders, and those with interaction indices < 1 are Non-Responders (Supplementary Table S1).

The GAG/DNA and interaction index of all cocultured pellets followed a normal data distribution and the Pearson’s *r* coefficient was -0.394 at a *p*-value of 0.006 (Figure 1D). The interaction index of the cocultures without PTHrP treatment and the GAG ratio of monoculture NC to monoculture BMSC showed a highly significant Pearson’s r coefficient of 0.79 × 10^–9^ (*p*-value = 1.99 × 10^–9^; Figure 1E). Interestingly, the GAG ratio of monoculture NC to monoculture BMSC for the “Responders” and “Non-Responders” distinctly clustered at opposite ends of the line of correlation (Figure 1E). For the Responders, there was no significant difference in GAG/DNA contents between the CC pellets (CC control) and cocultured pellets with PTHrP treatment (Figure 2A). However, the GAG/DNA content of monoculture NC was significantly higher by 4.3-fold (*p*-value = 3.5 × 10^–5^) than monoculture BM-MSC’s GAG/DNA content (Figure 2A). The interaction indices of all cocultured pellets with PTHrP treatment were not significantly different from coculture control pellet’s (Figure 2B). This suggests that PTHrP did not affect chondro-induction in cocultured pellets (Figure 2B). The ECM formation was also analyzed histologically, using Safranin-O, which stains sulfated GAG in a pinkish-red coloration (Rosenberg, 1971). Both monoculture NC and monoculture BM-MSC pellets showed positive Safranin-O staining. Although, like GAG/DNA measurements, BM-MSC pellets exhibited considerable donor-to-donor variability in Safranin-O staining (Figures 2C–N), cocultured pellets showed strong and consistent Safranin-O staining throughout the entire ECM (Figures 2C–N). No considerable differences were observed in Safranin-O staining when cocultured pellets were treated with increasing levels of PTHrP (Figures 2F,G,L–N), which again, suggests that PTHrP did not affect sulfated GAG matrix formation.

**FIGURE 3:**
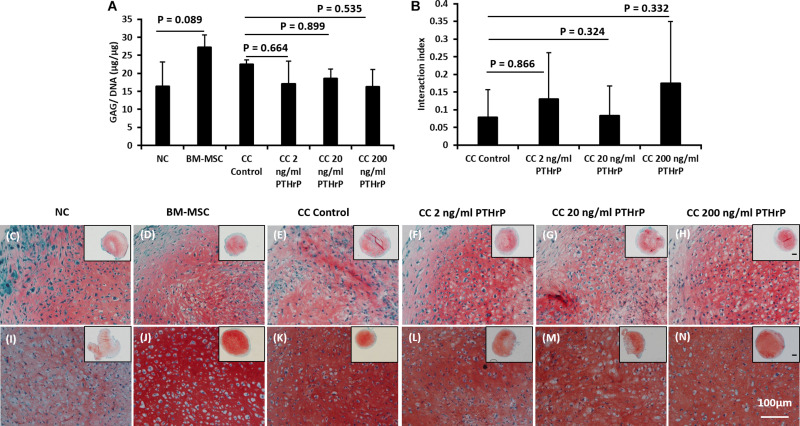
PTHrP does not affect Safranin-O staining in cocultured pellets with interaction index < 1 (i.e., Non-Responders). After 3 weeks of culture in chondrogenic media, pellets were fixed, embedded, and sectioned for histology. **(A)** The GAG per DNA contents of the monoculture and cocultured pellets associated with interaction index > 1. **(B)** The interaction index of cocultured pellets. **(C–H)** Safranin O staining of pellets formed from 250,000 cells and **(I–N)** safranin staining of pellets formed from 500,000 cells. The main and inset images are at 200x and 100x optical magnification, respectively. Scale bar are 100 μm. Statistical significance was determined using one-way ANOVA with Tukey’s HSD *post hoc* test (three donors in total; two donors in duplicates at 250,000 cells per pellet and one donor in duplicates at 500,000 cells per pellet; *n* = 6; ^∗∗∗^*p* < 0.001) (*n* = 6; *p* > 0.05).

For the Non-Responders, there was no significant difference between the GAG/DNA contents of CC control and cocultured pellets with PTHrP (Figure 3A). Moreover, unlike in the case of Responders, there was no statistical difference between the GAG/DNA contents of monoculture NC and monoculture BM-MSC (Figure 3A). All the interaction indices of cocultured pellets with PTHrP treatment were < 1 and not statistically different from CC Control pellets (Figure 3B). Furthermore, the ECM of all the Non-Responders cocultured pellets and their corresponding monoculture NC and BM-MSC were strongly positive for Safranin O staining (Figures 3C–N).

### PTHrP Treatment Reduced Expression of *COL10A1* in Cocultured Pellets

**FIGURE 4:**
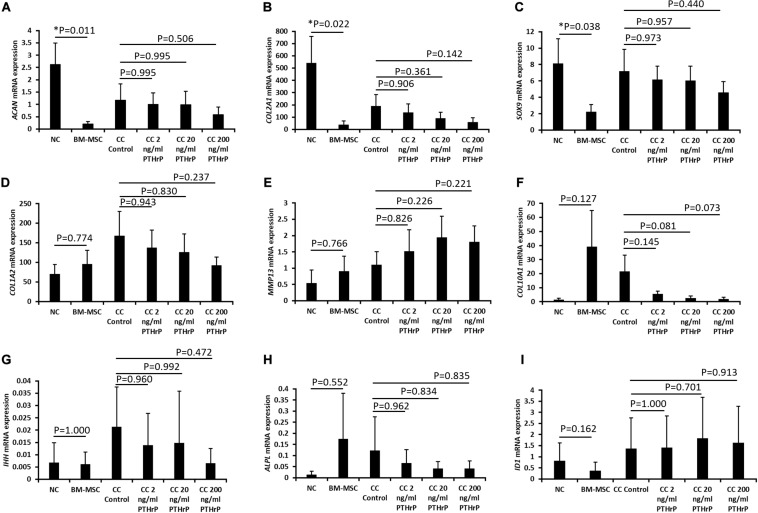
PTHrP affected the expression of *COL10A1* without other markers of chondrocyte hypertrophy in cocultured pellets with interaction indices > 1 (i.e., Responders). Pellets were lyzed in TRIzol and the RNA was extracted. cDNA was synthesized and gene expression was analyzed by qPCR. The expression of chondrogenic genes: **(A)** *ACAN*, **(B)** *COL2A1*, **(C)** *SOX9*, **(D)** *COL1A2*, **(E)** *MMP13*, **(F)** *COL10A1*, **(G)** *IHH*, **(H)** *ALPL*, and **(I)** *ID1* were examined. Expression of each gene was normalized to *B2M*, *HMBS*, and *YWHAZ* and expressed as the 2^– Δ^ ^Δ^ ^*Ct*^ value, shown on the *y*-axis. The *x*-axis indicates the sample and treatment. Statistical significance was determined using one-way ANOVA with Games-Howell *post hoc* test of unequal variances assumed (three donors in total; two donors in duplicates at 250,000 cells per pellet and one donor in duplicates at 500,000 cells per pellet; *n* = 6; *p < 0.05).

**FIGURE 5:**
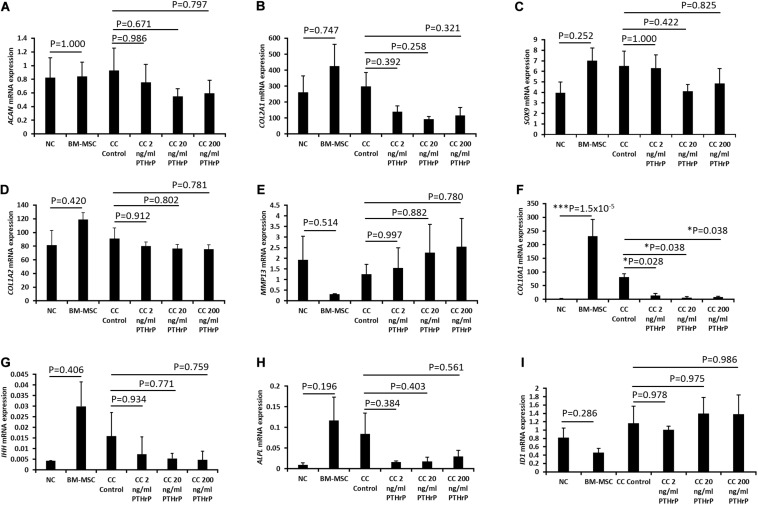
PTHrP affected the expression of *COL10A1* without other markers of chondrocyte hypertrophy in cocultured pellets with interaction indices < 1 (i.e., Non-Responders). Pellets were lyzed in TRIzol and the RNA was extracted. cDNA was synthesized and gene expression was analyzed by qPCR. The expression of chondrogenic genes: **(A)** *ACAN*, **(B)** *COL2A1*, **(C)** *SOX9*, **(D)** *COL1A2*, **(E)** *MMP13*, **(F)** *COL10A1*, **(G)** *IHH*, **(H)** *ALPL*, and **(I)** *ID1* were examined. Expression of each gene was normalized to *B2M*, *HMBS*, and *YWHAZ* and expressed as the 2^– Δ^ ^Δ^ ^*Ct*^ value, shown on the *y*-axis. The *x*-axis indicates the sample and treatment. Statistical significance was determined using one-way ANOVA with Games-Howell *post hoc* test of unequal variances assumed (three donors in total; two donors in duplicates at 250,000 cells per pellet and one donor in duplicates at 500,000 cells per pellet; *n* = 6; *p < 0.05, ***p < 0.001).

The expressions of chondrogenic genes (*ACAN*, *COL2A1*, and *SOX9*), fibrogenic gene (*COL1A2*), and hypertrophic and bone development associated genes (*COL10A1*, *MMP13*, *IHH*, *ALPL*, and *ID1*) were measured in each pellet sample by qPCR and expressed using the delta delta CT method (2^–Δ^ ^Δ^ ^*CT*^) (Figures 4, 5). For the Responders, the expression of *ACAN* was significantly higher in monoculture NC pellets relative to monoculture BM-MSC (*p* = 0.011; Figure 4A). *ACAN* expression in cocultured pellets was not significantly affected by PTHrP treatment (Figure 4A).

For the Responders, type I (*COL1A2*) and type II (*COL2A1*) collagens were expressed in monoculture NC and monoculture BM-MSC pellets. *COL1A2* showed no significant difference between monoculture NC and monoculture BM-MSC pellets (*p* > 0.05; Figure 4D). But *COL2A1* showed significantly higher expression in monoculture NC pellets relative to monoculture BM-MSC pellets (*p*-value = 0.022; Figure 4B). Both *COL1A2* and *COL2A1* were highly expressed in cocultured pellets and there was no significant difference between their expression in control cocultured pellets and those treated with PTHrP (*p* > 0.05; Figures 4B,D). The expression of *SOX9* was significantly higher in monoculture NC pellets relative to monoculture BM-MSC pellets (*p*-value = 0.038; Figure 4C). But there were no significant differences between control cocultured pellets and cocultured pellets with PTHrP (*p* > 0.05; Figure 4C). The expression of *COL10A1* was highly variable in monoculture BM-MSC and there was no significant difference in its expression in monoculture NC relative to monoculture BM-MSC (*p* > 0.05; Figure 4F). Furthermore, *COL10A1* expressions in control cocultured pellets and cocultured pellets with PTHrP were not significantly different (*p* > 0.05; Figure 4F). But there was a trend of decline in *COL10A1* expression with increasing PTHrP concentration. The latter is supported by a decreasing *p*-value (Figure 4E). *MMP13* showed similar expression levels in monoculture NC and monoculture BM-MSC pellets (*p*-value = 0.766; Figure 4E). Cocultured pellets showed slightly elevated levels of *MMP13* relative to both monoculture pellets and expression appeared to increase with PTHrP treatment (Figure 4E). There was no significant difference in *MMP13* expression between control cocultured pellets and cocultured pellets treated with PTHrP (*p* > 0.05; Figure 4E). The expression of *IHH* was variable in all pellets and more so in cocultured pellets (Figure 4G). There was no significant difference in its expression between monoculture NC and monoculture BM-MSC pellets and neither were there any differences in its expression between control cocultured pellets and cocultured pellets with PTHrP (*p* > 0.05; Figure 4G). The expression of *ALPL* was highly variable in monoculture of BM-MSC and in control cocultured pellets (Figure 4H). There was no statistical difference in the expression of IHH between monocultures of NC and BM-MSC (*p* > 0.05; Figure 4H). Additionally, there was no significant difference between the expression of *ALPL* in control cocultured and cocultured pellets with PTHrP treatment (*p* > 0.05; Figure 4H). The expression of ID1 was not different between monocultures of NC and BM-MSC (*p* > 0.05; Figure 4I). Similarly, the expression of ID1 was not different between control cocultured and cocultured pellets with PTHrP concentration (*p* > 0.05; Figure 4I).

For the Non-Responder cocultured pellets and their monoculture NC and BM-MSC pellets, except for *COL10A1* expression, all the other genes investigated were not differentially expressed between compared pellet groups (Figure 5). The expression of *COL10A1* was significantly higher in monocultures of BM-MSC relative to monoculture of NC (*p* = 1.5 × 10^–5^; Figure 5F). Furthermore, the expression of *COL10A1* in control cocultured pellets was significantly higher than in all cocultured pellets with PTHrP treatment (*p* < 0.05; Figure 5F).

### PCA Revealed a Non-hypertrophic and Hypertrophic *in vitro* Chondrogenesis in Cocultured Pellets

PCA was used to summarize the relationship between exogenous PTHrP dose and the measured molecular expression of *ACAN*, *COL1A2*, *COL2A1*, *SOX9*, *COL10A1*, *MMP13*, *IHH*, *ALPL*, and *ID1* during *in vitro* chondrogenesis of cocultured pellets of NC and BM-MSC. Regardless of whether the cocultures of NC and BM-MSC resulted in interaction indices > 1, the distribution of these variables proved normal by Shapiro–Wilk statistical test.

Pearson’s bivariate correlation statistics demonstrated significant positive correlation between non-hypertrophy chondrogenic genes (i.e., *ACAN*, *COL2A1*, and *SOX9*) with Pearson’s correlation (*r*) coefficients > 0.6 (Supplementary Table S4) for the monocultured and cocultured pellets associated with interaction indices > 1 (i.e., Responders). The expression of *ACAN* and *COL10A1*, a marker of chondrocyte hypertrophy, correlated negatively with significance (Pearson’s *r* of -0.375; *p*-value = 0.012; Supplementary Table S4). *COL10A1* and *IHH* showed no significant relation (Pearson’s *r* of -0.002; *p*-value = 0.495; Supplementary Table S3). Moreover, *SOX9* (a transcription factor for chondrogenesis) and *COL10A1* correlated negatively (Pearson’s *r* of -0.319; *p*-value = 0.029; Supplementary Table S3). The Kaiser-Meyer-Olkin (KMO) measure of sampling adequacy of the variables was 0.609 (Supplementary Table S5) and proved adequate to perform PCA (Cerny and Kaiser, 1977). The PCA resulted in a two-principal component (PC) solution; PC1 and PC2 (Supplementary Figure S1). PC1 and PC2, respectively, accounted for ∼ 30.9 and ∼27% of the total variance with respective eigenvalues of 3.09 and 2.73 (Supplementary Table S6). PC1 correlated in this order with *ACAN*, *COL2A1*, *SOX9*, *COL10A1*, *COL1A2*, *ID1*, *ALPL*, *MMP13*, and *IHH* (Table 1). PC2 ranked in this order *MMP13*, *COL1A2*, *ID1*, *IHH*, *ALPL*, *SOX9*, *COL2A1*, *COL10A1*, and *ACAN* (Table 1).

**TABLE 1:** Principal component analysis (PCA) correlation matrix for Responders.

Variables	PC1	PC2
PTHrP	–0.261	0.189
ACAN	0.966	–0.020
COL1A2	0.148	0.814
COL2A1	0.900	–0.205
COL1 0A1	–0.511	–0.097
SOX9	0.835	0.271
IHH	0.031	0.663
MMP13	–0.173	0.883
ALPL	–0.188	0.440
ID1	0.316	0.787

*Extraction method: principal component analysis. Rotation method: Varimax with Kaiser normalization.*

For monoculture and cocultured pellets with interaction indices < 1 or Non-Responders, *SOX9* and *COL10A1* correlated positively with high significance (Pearson’s *r* of 0.577; *p*-value = 0.006; Supplementary Table S7). *ACAN* and *COL10A1* showed no significant relationship (Pearson’s *r* of 0.325; *p*-value = 0.094; Supplementary Table S7). *COL10A1* and *IHH* showed highly significant positive correlation (Pearson’s *r* of 0.664; *p*-value = 0.001; Supplementary Table S7). The KMO measure of sampling adequacy was 0.647 and adequate to perform PCA (Supplementary Table S8). The PCA resulted in PC1 and PC2 (Supplementary Figure S2). PC1 and PC2, respectively, accounted for ∼57% and ∼12 of the total variances with eigenvalues of 5.69 and 1.24 (Supplementary Table S9). PC1 correlated in this order *COL1A2* > *COL10A1* > *COL2A 1* > *IHH* > *ALPL* > *ID1* > *MMP13* > *ACAN* (Table2). PC2’s order of correlation was *ACAN* > *MMP13* > *SOX9* > *ALPL* > *IHH* > *ID1* > *COL2A1* > *COL10A1* > *COL1A2* (Table 2).

**TABLE 2:** Principal component analysis (PCA) correlation matrix for Non-Responders.

Variables	PC1	PC2
PTHrP	–0.352	–0.250
ACAN	0.107	0.937
COL1A2	0.932	0.022
COL2A1	0.761	0.341
COL10A1	0.846	0.282
SOX9	0.353	0.798
IHH	0.687	0.528
MMP13	–0.311	–0.807
ALPL	0.680	0.559
1D1	–0.508	–0.449

*Extraction method: principal component analysis. Rotation method: Varimax with Kaiser normalization.*

### PTHrP Treatment Affected COL X but Not COL I and COL II Immunofluorescence (IF) in Cocultured Pellets

**FIGURE 6:**
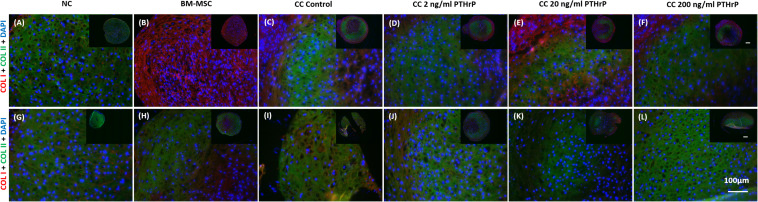
Type I and type II collagen immunofluorescence in pellets treated with PTHrP. After 3 weeks of culture in chondrogenic media pellets were fixed in 10% v/v formalin, sectioned, and probed with antibodies to type I collagen (COL I) and type II collagen (COL II). Sections were imaged by fluorescent microscopy. The cell source nasal chondrocytes (NC), bone marrow-derived mesenchymal stem cells (BM-MSC), or coculture (CC) and PTHrP treatment is indicated at the top of the images. COL I is shown in red, COL II is shown in green, and DAPI is shown in blue in the merged images. **(A–F)** Immunofluorescence images of pellets associated with interaction indices > 1 (i.e., Responders). **(G–L)** Immunofluorescence images from pellets associated with interaction indices < 1 (i.e., Non-Responders). All pellets here were formed from 250,000 cells. All main and inset images are shown at 200x and 100x optical zoom, respectively. The scale bars are 100 μm.

Pellets were fixed, embedded, and sectioned for IF of types I (COL I), II (COL II), and X (COL X) collagen proteins. Both COL I and COL II IF was consistently present throughout the entirety of NC and BMSC pellets (Figure 6). Control cocultured pellets also showed consistent IF of both COL I and COL II. Cocultured pellets treated with increasing concentrations of PTHrP also showed consistent COL I and COL II IF, with no observable differences relative to control cocultured pellets (Figure 6). But there were notable differences in the IF of COLI and COL II between the monoculture pellets of BM-MSC of the Responders (Figures 6A–F) and Non-Responders (Figures 6G–L). The Responders had a strong IF for COL I and low to IF for COL II in the monocultured pellets of BM-MSC (Figure 6B). In contrast, the Non-Responders showed intense IF for COL I and COL II (Figure 6H).

**FIGURE 7:**
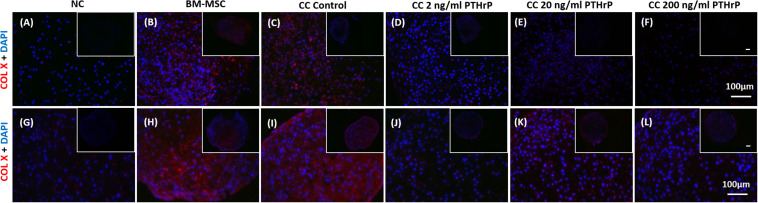
COL X immunofluorescence declined in pellets treated with PTHrP *in vitro*. After 3 weeks of culture in chondrogenic media pellets were fixed in 10% v/v formalin, sectioned, and probed with antibodies to type X collagen. Sections were imaged by fluorescent microscopy. The cell source nasal chondrocytes (NC), bone marrow-derived mesenchymal stem cells (BM-MSC), or coculture (CC) and PTHrP treatment is indicated at the top of the images. COL X is shown in red and DAPI is shown in blue. **(A–F)** Immunofluorescence images of pellets associated with interaction indices > 1 (i.e., Responders). **(G–L)** Immunofluorescence images from pellets associated with interaction indices < 1 (i.e., Non-Responders). All pellets here were formed from 250,000 cells. All main and inset images are shown at 200x and 100x optical zoom, respectively. The scale bars are100 μm.

Nasal chondrocyte pellets showed little to no detectable COLX by IF (Figure 7). In contrast, BM-MSC pellets revealed the presence of COL X throughout the entire pellet, with strong IF for COL X protein (Figure 5). The intensity of COL X seemed more intense in the monocultured pellets of BM-MSC (Figures 7B,H) and control cocultured pellets (Figures 7C,I), and perhaps more so in the monocultured pellets (Figure 7H) and control cocultured pellets (Figure 7I) of the Non-Responders. Consistent with the gene expression analysis, COL X IF was considerably reduced in cocultured pellets treated with PTHrP. COL X IF is reduced at all levels of PTHrP treatment, with little detectable signal observed around the periphery of the pellets (Figures 7D–F,J–L).

### Implanted Cocultured Pellets Had Interaction Indices > 1 and PTHrP Did Not Affect Chondro-Induction

**FIGURE 8:**
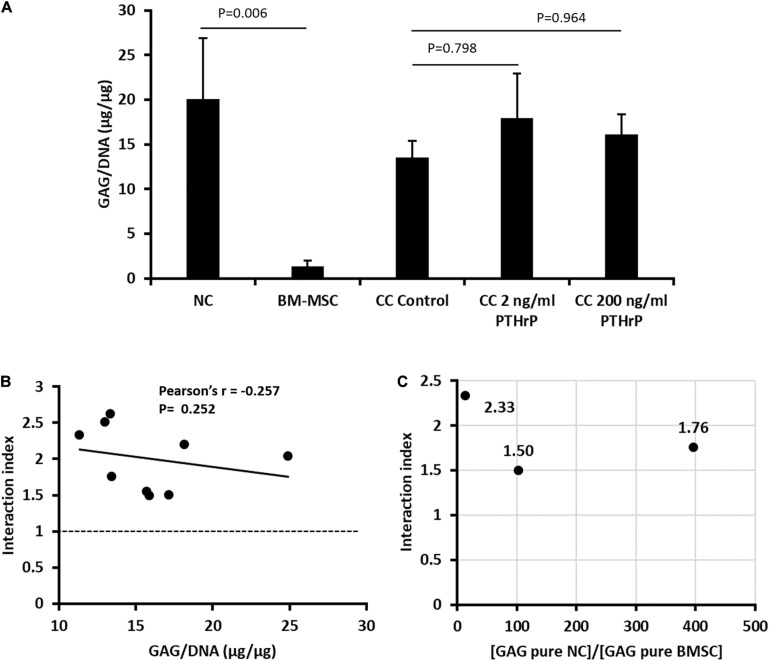
PTHrP treated and *in vivo* implanted cocultured pellets had interaction indices > 1. Implanted pellet cultures were formed from 5 × 10^5^ cells from nasal chondrocytes (NC), bone marrow-derived mesenchymal stem cells (BM-MSC), or cocultures consisting of NC and BM-MSC at a 1:3 ratio (CC) (see Supplementary Table S2). Pellets were cultured in chondrogenic media for three weeks without (control) or with PTHrP at 2 or 200 ng/mL. After 3 weeks of culture, the pellets were analyzed for: **(A)** GAG per DNA; **(B)** Pearson’s bivariate correlation of the interaction indices [based on calculations from Acharya et al. (3)] and GAG per DNA contents of cocultured pellets with or without PTHrP treatments. **(C)** Distribution of the interaction indices of control cocultured pellets with the ratio of GAG contents of monoculture (pure) of NC to GAG content of monoculture (pure) BM-MSC. Three donors without replicates (*n* = 3, *p* > 0.05).

Prior to implantation of monocultured pellets of NC and BM-MSC, and cocultured pellets of NC and BM-MSC, the GAG and DNA contents of the pellets were determined (Figure 8A). The GAG per DNA contents of the monocultured pellets of NC were significantly higher than those of the BM-MSC pellets (Figure 8A). PTHrP did not affect the GAG per DNA content of the cocultured pellets (Figure 8A). The interaction indices of all cocultured pellets were ≥ 1.5 (Figure 8B and Supplementary Table S2). There was slight inverse relationship between the interaction indices and GAG per DNA contents of the cocultured pellets, but this was not significant (Pearson’s *r* = -0.252; *P* = 0.252; Figure 8B). The ratio of the GAG contents of NC monocultured pellets to those of BM-MSC monocultured pellets was consistently > 1 (Figure 8C). These findings were consistent with cocultured pellets designated as Responders in Figure 1E.

### PTHrP Treatment Did Not Affect COL I and COL II Immunofluorescence (IF) in Implanted Cocultured Pellets

**FIGURE 9:**
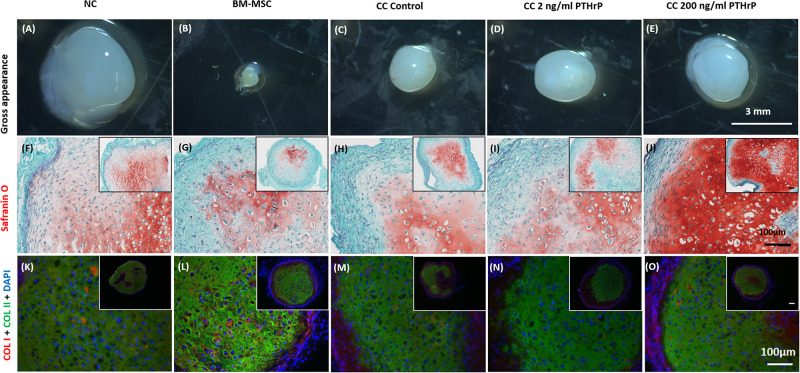
Safranin O staining, types I (COL I) and II (COL II) collagen immunofluorescence in pellets treated with PTHrP after 3 weeks of implantation in nude mice. After 3 weeks of implantation in nude mice, pellets were harvested, photographed, fixed in 10% formalin, sectioned, subjected to Safranin O staining, and probed with fluorescent antibodies to types I (COL I) and II (COL II). The cell source nasal chondrocytes (NC), bone marrow-derived mesenchymal stem cells (BM-MSC), or coculture (CC) and PTHrP (2 and 200 ng/ml) treatment is indicated at the top of the images. **(A–E)** Gross appearance of the pellets. **(F–J)** Safranin O staining histological images. **(K–O)** COLI is shown in red, COLII is shown in green, and DAPI is shown in blue in the merged images. All main and inset images are shown at 200x and 100x optical zoom, respectively. The scale bars are 100 μm.

In order to evaluate the propensity for tissues to calcify *in vivo*, pellet constructs were subcutaneously implanted into athymic nude CD-1 mice for 3 weeks. After 3 weeks, the pellets were explanted for gross appearance assessment and histological analysis (Figure 9). The explants appeared glassy white (Figures 9A–E). The largest and smallest pellets were, respectively, monocultured NC and BM-MSC pellets (Figures 9A,B, respectively). All pellets were positive for Safranin O staining albeit with some variability in intensity. The cocultured pellet with 200 ng/mL PTHrP treatment appeared the most intense (Figure 9J). Similarly, all pellets were positive for COL I and COL II (Figures 9K–O).

### PTHrP Treatment Reduced COL X Immunofluorescence (IF) in Implanted Cocultured Pellets

**FIGURE 10:**
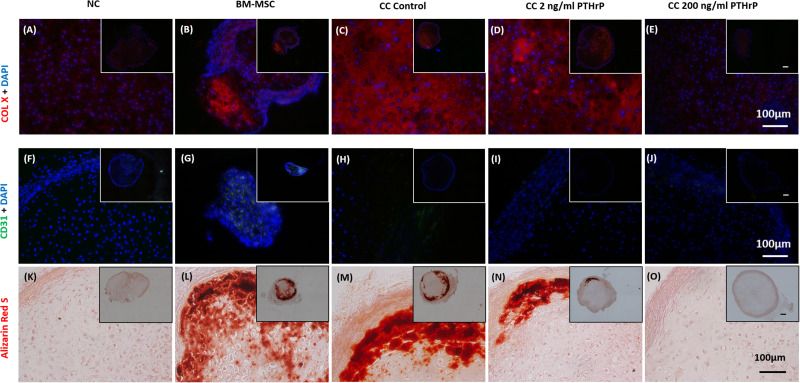
Alizarin Red S staining, COL X and CD31 immunofluorescence is reduced in pellets treated with PTHrP after 3 weeks of implantation in nude mice. After 3 weeks of implantation in nude mice, pellets were harvested, fixed in 10% formalin, sectioned, subjected to Alizarin Red S staining, and probed with fluorescent antibodies to COL X and CD31. Sections were imaged by brightfield/fluorescent microscopy. The cell source nasal chondrocytes (NC), bone marrow-derived mesenchymal stem cells (BM-MSC), or coculture (CC) and PTHrP treatment is indicated on top of the images. **(A–E)** COL X and DAPI are shown in blue in the merged images. **(F–J)** CD31 and DAPI are shown in green and blue, respectively, in the merged images. **(K–O)** Alizarin Red S staining histological images. All main and inset images are shown at 200x and 100x optical zoom, respectively. The scale bars are 100 μm.

Like the *in vitro* IF analysis, NC pellets revealed a low degree of COL X IF (Figure 10A), whereas BM-MSC pellets showed relatively intense COL X IF throughout most of the microtissue pellet (Figure 10B). Similarly, control cocultured pellets also showed a high degree of COL X IF throughout most of the pellet (Figure 10C). Treatment of cocultured pellets with 2 and 200 ng/mL of PTHrP prior to implantation appeared to reduce COL X IF after 3 weeks *in vivo* in a dose-dependent manner (Figures 10D,E). Little to no COL X IF was observed in cocultured pellets when treated with 200 ng/mL PTHrP prior to implantation (Figure 10E).

### PTHrP Treatment Reduced CD31 Immunofluorescence (IF) in Implanted Cocultured Pellets

Implanted pellets were also analyzed for CD31 expression by IF (Figures 10F–J), which revealed extensive CD31 expression in implanted BM-MSC samples throughout the entire pellet (Figure 10G). In contrast, CD31 IF was absent in implanted NC pellets (Figure 10F). Control cocultured pellets showed CD31 IF decorating the periphery of the pellets (Figure 10H). CD31 IF was reduced in cocultured pellets when pretreated with PTHrP prior to implantation (Figures 10I,J). This effect appeared to be dose-dependent, as cocultured pellets pretreated with 200 ng/mL PTHrP prior to implantation revealed no visible CD31 IF (Figure 10J).

### PTHrP Treatment Reduced Calcium Deposits in Implanted Cocultured Pellets

Alizarin Red S is a commonly used histological tool to stain calcium deposits (Puchtler et al., 1969). Implanted pellets were histologically analyzed for calcification using Alizarin Red S (Figures 10K–O). BM-MSC pellets showed extensive Alizarin Red S staining throughout the pellet, which suggested a high degree of calcification (Figure 10L). In contrast, NC pellets showed little to no Alizarin Red S staining (Figure 10K). Control cocultured pellets also revealed considerable Alizarin Red S staining throughout the pellet, particularly localized circumferentially (Figure 10M). Treatment of cocultured pellets prior to implantation appeared to reduce calcium deposits in a dose-dependent manner. Cocultured pellets treated with 2 ng/mL of PTHrP prior to implantation revealed reduced Alizarin Red S staining relative to control cocultured pellets (Figure 10N). Furthermore, cocultured pellets treated with an increased dosage of PTHrP (200 ng/mL) revealed no visible Alizarin Red S staining, suggesting an absence of calcium deposits (Figure 10O).

## Discussion

The present study is the first to demonstrate the feasibility of eradicating the *in vivo* calcification of engineered cartilage from cocultures of NC and BM-MSC by pre-treatment with PTHrP as a suppressor of hypertrophic differentiation of BM-MSC. The molecular, biochemical, and histological characteristics of the engineered cartilage were similar to cartilage engineered from monoculture NC, which has been reported in two first-in-human trials to repair cartilage defects in the knee and nose (Fulco et al., 2014; Mumme et al., 2016). Our findings offer the perspective that a small proportion of minimally expanded human NC from nasal septal cartilage can be used in conjunction with extensively expanded plastic-adherent cultured human BM-MSC to generate stable implantable 3D engineered cartilage grafts.

In considering treatments to improve the phenotypic stability of implanted engineered cartilage constructs, it is important to consider the potential effects on chondrogenic capacity and ECM formation. PTHrP did not affect GAG matrix production. In addition, when chondro-induction was calculated, it was found that PTHrP did not affect the synergistic effect that has been both previously reported of NC:BM-MSC cocultures (Acharya et al., 2012; Andrews et al., 2017) and observed in this study. In addition, this was further supported by *ACAN* gene expression, which showed no significant change with PTHrP treatments. *ACAN* encodes for aggrecan, a major GAG containing proteoglycan component of cartilage (Kiani et al., 2002).

While the motivation for this study was inspired by the reported chondro-induction of NC:BM-MSC cocultures as a strategy to enhance the functional ECM and quality of engineered cartilage graft, not all NC:BM-MSC cocultures in this study resulted in chondro-induction. The reason for the lack seemed to be associated with the ratio of *in vitro* chondrogenic capacity of the NC and BM-MSC of the cocultures. However, there is the possibility that the 1:3 ratio of the NC:BM-MSC cocultures of this study was not optimal to ensure constant chondro-induction. Regardless of the occurrence of chondro-induction, PTHrP did not affect the expression of a cohort of chondrogenic marker genes (i.e., *ACAN*, *COL2A1*, and *SOX9*) but suppressed the expression of hypertrophic marker, *COL10A1* and not *MMP-13*, *IHH*, *ALPL*, and *ID1* as other markers of chondrocyte hypertrophy (Figures 4, 5). The reason for this is unclear without further investigation but appears to indicate that the expression of *MMP-13* and the rest of the tested panel of markers of chondrocyte hypertrophy are uncoupled from *COL10A1* and modulated by alternative pathways (Brew et al., 2010; van der Kraan and van den Berg, 2012).

The alternative pathway may be the WNT signaling. WNT signaling has been implicated in hypertrophic differentiation of BM-MSC (Narcisi et al., 2015; Diederichs et al., 2019). Our data indirectly support this possibility given the fact that PTHrP did not suppress *MMP-13*, *IHH*, *ALPL*, and *ID1* but the WNT secretion inhibitor *N*-(6-methyl-2-benzothiazolyl)-2-[(3,4,6,7-tetrahydro-4-oxo-3-phenylthieno[3,2-d]pyrimidin-2-yl)thio]-acetamide (IWP2) did (Narcisi et al., 2015; Diederichs et al., 2019).

The potency of PTHrP in suppressing *COL10A1* seemed to be influenced by chondro-induction. In cocultures with chondro-induction, the suppression of *COL10A1* increased with PTHrP dose (Figure 4F). In contrast, the least tested PTHrP dose (i.e., 2 ng/mL) significantly suppressed *COL10A1* expression in the cocultures lacking chondro-induction (Figure 5F) but not in cocultures with chondro-induction (Figure 4F). The molecular basis of this nuance is unclear. But the PCA results of NC:BM-MSC cocultures lacking chondro-induction (i.e., Non-Responders) had hypertrophic differentiation in a higher ranking order relative to cocultures with chondro-induction (Tables 1, 2). Therefore, suggesting that the cocultures with higher expression of hypertrophic differentiation were more susceptible to PTHrP’s capacity to suppress *COL10A1*.

Because PTHrP did not affect ECM production, it is not precluded as a candidate to increase the stability of engineered cartilage constructs. Type X collagen provides structural framework for calcification and transition of hypertrophic cartilage tissue to bone (Kielty et al., 1985; Kirsch and von der Mark, 1991; von der Mark et al., 1992; Shen, 2005). In addition, it has been shown that *COL10A1* expression is increased in *in vitro* cocultured NC:BM-MSC constructs and once implanted in athymic nude mice, *in vivo* calcification results (Andrews et al., 2017). The reduction in transcription and translation of type X collagen observed with PTHrP treatment *in vitro* (Figures 4F, 5E, 7D–F,J–L), particularly after implantation (Figures 10A–E), suggests that PTHrP was effective in reducing hypertrophic differentiation in NC and BM-MSC cocultured constructs. Furthermore, reduction in COL X IF appears to be dose dependent as higher dose treatments appear to show further reduced COL X IF.

It has been shown that coculture cartilage constructs consisting of BM-MSC are prone to calcification and vascular invasion *in vivo* (Andrews et al., 2017). CD31 is a platelet endothelial cell adhesion molecule (Newman et al., 1990) that is commonly used as a marker for vascularization, as it demonstrates the presence of endothelial cells (Xie and Muller, 1996; Andrews et al., 2017). Calcification of hypertrophic cartilage *in vivo* is preceded by vascularization of the native tissue (Carlevaro et al., 2000; Hattori et al., 2010), as bone is heavily vascularized relative to cartilage (Maes, 2013). This was evident by the observed CD31 IF in BM-MSC pellets *in vivo*, which showed the prevalence of CD31 throughout most of the construct. While the control coculture constructs showed reduced CD31 IF relative to the BM-MSC constructs, CD31 was still present, which suggests that some vascularization occurred. Furthermore, the reduction in CD31 IF observed in treated coculture constructs suggests that PTHrP also reduced vascularization *in vivo*, which contributes to the stability of the constructs once implanted. Most significantly, after 3 weeks of implantation in nude mice, coculture constructs treated with 200 ng/mL of PTHrP prior to implantation showed no calcification, as demonstrated by absence of Alizarin Red S staining. In contrast, control coculture constructs revealed considerable Alizarin Red S staining after the 3 weeks of implantation, which is consistent with previous reports (Andrews et al., 2017). Again, this suggests that pre-treatment of coculture constructs with PTHrP, prior to implantation, improves the phenotypic stability of the constructs. In addition, while treatment with PTHrP at 2 ng/mL showed evidence of some calcification and 200 ng/mL treatment was enough to prevent any calcification from occurring. It is possible that doses between 2 and 200 ng/mL would be enough to prevent calcification *in vivo*, such as 20 ng/mL that we examined *in vitro* but failed to explore further in attempt to conserve animal numbers.

Our results demonstrate that the treatment of NC:BM-MSC coculture constructs with PTHrP prior to implantation suppressed *COL10A1* expression and type X collagen production and correlated with the lack of *in vivo* calcification. Overall, our results suggest that PTHrP can be used to increase the stability of tissue-engineered cartilage constructs consisting of BM-MSCs prior to implantation. However, the optimal dosage of PTHrP merits further investigation both *in vitro* and *in vivo.*

## Data Availability Statement

The raw data supporting the conclusions of this article will be made available by the authors, without undue reservation.

## Ethics Statement

The studies involving human tissue specimens were reviewed and approved by the University of Alberta’s Health Research Ethics Board. Written informed consent for participation was not required for this study in accordance with the national legislation and the institutional requirements. The animal study was reviewed and approved by the University of Alberta Animal Care and Use Committee.

## Author Contributions

MA-B conducted the bulk of the experiments and was responsible for data acquisition, analysis, and manuscript writing. YL performed the animal surgery with assistance from MK. MK was responsible for performing qRT-PCR and gene expression analysis. AM-S performed cell culture work. MO, KA, and HS were involved in human nasal septal cartilage procurement and in manuscript writing and review. AA conceived the study, supervised the study, performed PCA and other statistical analysis, and was responsible for writing and final review of the manuscript. All authors read and approved the final manuscript.

## Conflict of Interest

The authors declare that the research was conducted in the absence of any commercial or financial relationships that could be construed as a potential conflict of interest.

## References

[B1] Acharya C. Adesida A. Zajac P. Mumme M. Riesle J. Martin I. (2012). Enhanced chondrocyte proliferation and mesenchymal stromal cells chondrogenesis in coculture pellets mediate improved cartilage formation. *J. Cell Physiol.* 227 88–97. 10.1002/jcp.22706 22025108

[B2] Anderson-Baron M. Kunze M. Mulet-Sierra A. Adesida A. B. (2020). Effect of cell seeding density on matrix-forming capacity of meniscus fibrochondrocytes and nasal chondrocytes in meniscus tissue engineering. *FASEB J.* 34 5538–5551. 10.1096/fj.201902559R 32090374

[B3] Andrews S. H. J. Kunze M. Mulet-Sierra A. Williams L. Ansari K. Osswald M. (2017). Strategies to mitigate variability in engineering human nasal cartilage. *Sci. Rep.* 7:6490. 10.1038/s41598-017-06666-2 28747655 PMC5529506

[B4] Brew C. J. Clegg P. D. Boot-Handford R. P. Andrew J. G. Hardingham T. (2010). Gene expression in human chondrocytes in late osteoarthritis is changed in both fibrillated and intact cartilage without evidence of generalised chondrocyte hypertrophy. *Ann. Rheum. Dis.* 69 234–240. 10.1136/ard.2008.097139 19103633

[B5] Candrian C. Vonwil D. Barbero A. Bonacina E. Miot S. Farhadi J. (2008). Engineered cartilage generated by nasal chondrocytes is responsive to physical forces resembling joint loading. *Arthrit. Rheum.* 58 197–208. 10.1002/art.23155 18163475

[B6] Carlevaro M. F. Cermelli S. Cancedda R. Descalzi Cancedda F. (2000). Vascular endothelial growth factor (VEGF) in cartilage neovascularization and chondrocyte differentiation: auto-paracrine role during endochondral bone formation. *J. Cell Sci.* 113(Pt 1), 59–69. 10591625 10.1242/jcs.113.1.59

[B7] Caron M. M. Emans P. J. Surtel D. A. van der Kraan P. M. van Rhijn L. W. Welting T. J. (2015). BAPX-1/NKX-3.2 acts as a chondrocyte hypertrophy molecular switch in osteoarthritis. *Arthrit. Rheum.* 67 2944–2956. 10.1002/art.39293 26245691

[B8] Cerny B. A. Kaiser H. F. (1977). A Study of a measure of sampling adequacy for factor-analytic correlation matrices. *Multivar. Behav. Res.* 12 43–47. 10.1207/s15327906mbr1201_3 26804143

[B9] Cooke M. E. Allon A. A. Cheng T. Kuo A. C. Kim H. T. Vail T. P. (2011). Structured three-dimensional co-culture of mesenchymal stem cells with chondrocytes promotes chondrogenic differentiation without hypertrophy. *Osteoarthrit. Cartil.* 19 1210–1218. 10.1016/j.joca.2011.07.005 21816228 PMC3188316

[B10] Diederichs S. Tonnier V. März M. Dreher S. I. Geisbüsch A. Richter W. (2019). Regulation of WNT5A and WNT11 during MSC in vitro chondrogenesis: WNT inhibition lowers BMP and hedgehog activity, and reduces hypertrophy. *Cell. Mol. Life Sci.* 76 3875–3889. 10.1007/s00018-019-03099-0 30980110 PMC11105731

[B11] Fischer J. Dickhut A. Rickert M. Richter W. (2010). Human articular chondrocytes secrete parathyroid hormone-related protein and inhibit hypertrophy of mesenchymal stem cells in coculture during chondrogenesis. *Arthrit. Rheum.* 62 2696–2706. 10.1002/art.27565 20496422

[B12] Fulco I. Miot S. Haug M. D. Barbero A. Wixmerten A. Feliciano S. (2014). Engineered autologous cartilage tissue for nasal reconstruction after tumour resection: an observational first-in-human trial. *Lancet* 384 337–346. 10.1016/s0140-6736(14)60544-4 24726477

[B13] Hattori T. Muller C. Gebhard S. Bauer E. Pausch F. Schlund B. (2010). SOX9 is a major negative regulator of cartilage vascularization, bone marrow formation and endochondral ossification. *Development* 137 901–911. 10.1242/dev.045203 20179096

[B14] Kafienah W. Mistry S. Dickinson S. C. Sims T. J. Learmonth I. Hollander A. P. (2007). Three-dimensional cartilage tissue engineering using adult stem cells from osteoarthritis patients. *Arthrit. Rheum.* 56 177–187. 10.1002/art.22285 17195220

[B15] Kiani C. Chen L. Wu Y. J. Yee A. J. Yang B. B. (2002). Structure and function of aggrecan. *Cell Res.* 12 19–32. 10.1038/sj.cr.7290106 11942407

[B16] Kielty C. M. Kwan A. P. Holmes D. F. Schor S. L. Grant M. E. (1985). Type X collagen, a product of hypertrophic chondrocytes. *Biochem. J.* 227 545–554. 10.1042/bj2270545 4004779 PMC1144874

[B17] Kirsch T. von der Mark K. (1991). Ca2+ binding properties of type X collagen. *FEBS Lett.* 294 149–152. 10.1016/0014-5793(91)81363-d 1743285

[B18] Kronenberg H. M. (2003). Developmental regulation of the growth plate. *Nature* 423 332–336. 10.1038/nature01657 12748651

[B19] Livak K. J. Schmittgen T. D. (2001). Analysis of relative gene expression data using real-time quantitative PCR and the 2(-Delta Delta C(T)) Method. *Methods* 25 402–408. 10.1006/meth.2001.1262 11846609

[B20] Maes C. (2013). Role and regulation of vascularization processes in endochondral bones. *Calcif. Tissue Int.* 92 307–323. 10.1007/s00223-012-9689-z 23292135

[B21] Mannstadt M. Juppner H. Gardella T. J. (1999). Receptors for PTH and PTHrP: their biological importance and functional properties. *Am. J. Physiol.* 277 F665–F675. 10.1152/ajprenal.1999.277.5.F665 10564229

[B22] Mumme M. Barbero A. Miot S. Wixmerten A. Feliciano S. Wolf F. (2016). Nasal chondrocyte-based engineered autologous cartilage tissue for repair of articular cartilage defects: an observational first-in-human trial. *Lancet* 388 1985–1994. 10.1016/S0140-6736(16)31658-0 27789021

[B23] Narcisi R. Cleary M. A. Brama P. A. J. Hoogduijn M. J. Tüysüz N. Berge D. (2015). Long-term expansion, enhanced chondrogenic potential, and suppression of endochondral ossification of adult human MSCs via WNT signaling modulation. *Stem Cell Rep.* 4 459–472. 10.1016/j.stemcr.2015.01.017 25733021 PMC4375944

[B24] Newman P. J. Berndt M. C. Gorski J. White G. C. II Lyman S. Paddock C. (1990). PECAM-1 (CD31) cloning and relation to adhesion molecules of the immunoglobulin gene superfamily. *Science* 247 1219–1222. 10.1126/science.1690453 1690453

[B25] Paschos N. K. Brown W. E. Eswaramoorthy R. Hu J. C. Athanasiou K. A. (2015). Advances in tissue engineering through stem cell-based co-culture. *J. Tissue Eng. Regen. Med.* 9 488–503. 10.1002/term.1870 24493315

[B26] Pelttari K. Pippenger B. Mumme M. Feliciano S. Scotti C. Mainil-Varlet P. (2014). Adult human neural crest-derived cells for articular cartilage repair. *Sci. Transl. Med.* 6:251ra119. 10.1126/scitranslmed.3009688 25163479

[B27] Pelttari K. Winter A. Steck E. Goetzke K. Hennig T. Ochs B. G. (2006). Premature induction of hypertrophy during in vitro chondrogenesis of human mesenchymal stem cells correlates with calcification and vascular invasion after ectopic transplantation in SCID mice. *Arthrit. Rheum.* 54 3254–3266. 10.1002/art.22136 17009260

[B28] Provot S. Kempf H. Murtaugh L. C. Chung U. I. Kim D. W. Chyung J. (2006). Nkx3.2*/Bapx*1 acts as a negative regulator of chondrocyte maturation. *Development* 133 651–662. 10.1242/dev.02258 16421188

[B29] Puchtler H. Meloan S. N. Terry M. S. (1969). On the history and mechanism of alizarin and alizarin red S stains for calcium. *J. Histochem. Cytochem.* 17 110–124. 10.1177/17.2.110 4179464

[B30] Ripmeester E. G. J. Timur U. T. Caron M. M. J. Welting T. J. M. (2018). Recent insights into the contribution of the changing hypertrophic chondrocyte phenotype in the development and progression of osteoarthritis. *Front. Bioeng. Biotechnol.* 6:18. 10.3389/fbioe.2018.00018 29616218 PMC5867295

[B31] Rosenberg L. (1971). Chemical basis for the histological use of safranin O in the study of articular cartilage. *J. Bone Joint Surg. Am.* 53 69–82. 10.2106/00004623-197153010-00007 4250366

[B32] Schmittgen T. D. Livak K. J. (2008). Analyzing real-time PCR data by the comparative C(T) method. *Nat. Protoc.* 3 1101–1108. 10.1038/nprot.2008.73 18546601

[B33] Scotti C. Osmokrovic A. Wolf F. Miot S. Peretti G. M. Barbero A. (2012). Response of human engineered cartilage based on articular or nasal chondrocytes to interleukin-1beta and low oxygen. *Tissue Eng. Part A* 18 362–372. 10.1089/ten.tea.2011.0234 21902467 PMC3267974

[B34] Shen G. (2005). The role of type X collagen in facilitating and regulating endochondral ossification of articular cartilage. *Orthod. Craniofac. Res.* 8 11–17. 10.1111/j.1601-6343.2004.00308.x 15667640

[B35] Solchaga L. A. Penick K. Goldberg V. M. Caplan A. I. Welter J. F. (2010). Fibroblast growth factor-2 enhances proliferation and delays loss of chondrogenic potential in human adult bone-marrow-derived mesenchymal stem cells. *Tissue Eng. Part A* 16 1009–1019. 10.1089/ten.tea.2009.0100 19842915 PMC2862658

[B36] Tay A. G. Farhadi J. Suetterlin R. Pierer G. Heberer M. Martin I. (2004). Cell yield, proliferation, and postexpansion differentiation capacity of human ear, nasal, and rib chondrocytes. *Tissue Eng.* 10 762–770. 10.1089/1076327041348572 15265293

[B37] van der Kraan P. M. van den Berg W. B. (2012). Chondrocyte hypertrophy and osteoarthritis: role in initiation and progression of cartilage degeneration? *Osteoarthrit. Cartil.* 20 223–232. 10.1016/j.joca.2011.12.003 22178514

[B38] von der Mark K. Kirsch T. Nerlich A. Kuss A. Weseloh G. Gluckert K. (1992). Type X collagen synthesis in human osteoarthritic cartilage. Indication of chondrocyte hypertrophy. *Arthrit. Rheum.* 35 806–811. 10.1002/art.1780350715 1622419

[B39] Weiss S. Hennig T. Bock R. Steck E. Richter W. (2010). Impact of growth factors and PTHrP on early and late chondrogenic differentiation of human mesenchymal stem cells. *J. Cell Physiol.* 223 84–93. 10.1002/jcp.22013 20049852

[B40] Wu L. Leijten J. C. Georgi N. Post J. N. van Blitterswijk C. Karperien M. (2011). Trophic effects of mesenchymal stem cells increase chondrocyte proliferation and matrix formation. *Tissue Eng. Part A* 17 1425–1436. 10.1089/ten.tea.2010.0517 21247341

[B41] Xie Y. Muller W. A. (1996). Fluorescence in situ hybridization mapping of the mouse platelet endothelial cell adhesion molecule-1 (PECAM1) to mouse chromosome 6, region F3-G1. *Genomics* 37 226–228. 10.1006/geno.1996.0546 8921400

