# Supplementary material for: Suppression of Hypertrophy During in vitro Chondrogenesis of Cocultures of Human Mesenchymal Stem Cells and Nasal Chondrocytes Correlates With Lack of in vivo Calcification and Vascular Invasion
Source: Front Bioeng Biotechnol. 2021 Jan 5;8:572356. doi: 10.3389/fbioe.2020.572356 (PMC7813892; doi:10.3389/fbioe.2020.572356)
Supplement: Supplementary file 2 [file Table_2.DOCX]

**
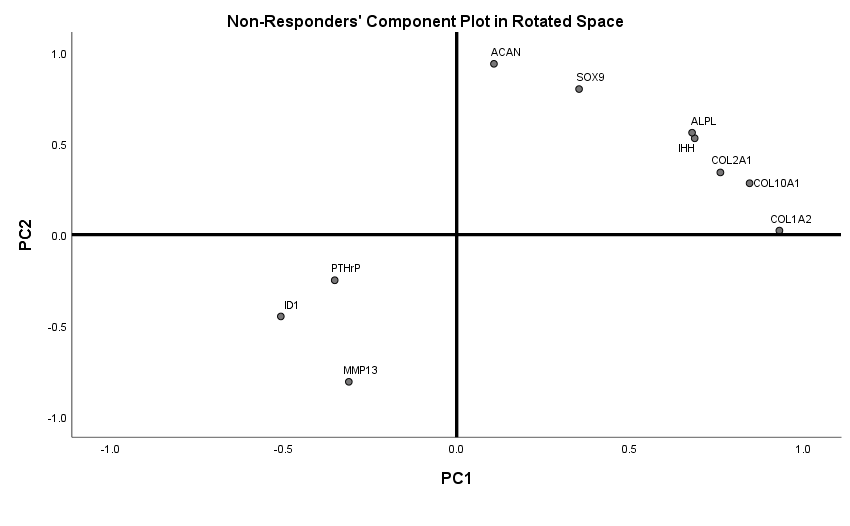
**

**Supplementary Figure 2**. **A varimax rotated plot of a two-component solution of principal component analysis of the correlation of PTHrP concentration and measured molecular gene variables.** PTHrP dose and the measured molecular expression of *ACAN, COL1A2, COL2A1, SOX9, COL10A1, MMP13, IHH, ALPL and ID1* during *in vitro* chondrogenesis of cocultured pellets of NC and BM-MSC. Principal component 1 is on the x-axis and principal component 2 is on the y-axis. The position of each variable on the plot indicates the degree to which it loads on the two different principal components.
