# Supplementary material for: Suppression of Hypertrophy During in vitro Chondrogenesis of Cocultures of Human Mesenchymal Stem Cells and Nasal Chondrocytes Correlates With Lack of in vivo Calcification and Vascular Invasion
Source: Front Bioeng Biotechnol. 2021 Jan 5;8:572356. doi: 10.3389/fbioe.2020.572356 (PMC7813892; doi:10.3389/fbioe.2020.572356)
Supplement: Supplementary file 3 [file Table_3.DOCX]

**Supplementary Table S1.** Human nasal chondrocytes (NC) and bone marrow mesenchymal stem cells (BM-MSC) coculture paring information with anonymized donor information related to only *in vitro* study

| **NC**  **donor** | **NC**  **(sex, age)** | **NC**  **CPD** | **BM-MSC**  **donor** | **BM-MSC**  **(sex, age)** | **BM-MSC**  **CPD** | **Mean control interaction index** | **Mean**  **2 ng/ml PTHrP**  **interaction**  **index** | **Mean**  **20 ng/ml PTHrP interaction index** | **Mean**  **200 ng/ml PTHrP interaction index** |
| --- | --- | --- | --- | --- | --- | --- | --- | --- | --- |
| NC167 | f, 34 | 3.85 | mBM323 | f, 35 | 17.93 | *^a^1.21* | *^a^1.49* | *^a^1.39* | *^a^1.50* |
| NC169 | f, 21 | 3.36 | mBM340 | f, 59 | 10.39 | *^a^1.13* | *^a^1.33* | *^a^1.29* | *^a^1.23* |
| NC144 | f, 36 | 7.66 | mBM334 | f, 40 | 13.46 | *^b^0.87* | *^b^0.83* | *^b^0.67* | *^b^0.45* |
| NC138 | m, 52 | 5.89 | mBM341 | m, 35 | 14.72 | *^b^0.76* | *^b^0.63* | *^b^0.61* | *^b^0.74* |
| *NC147 | m, 29 | 6.80 | *mBM345 | m, 58 | 13.55 | *^a^1.64* | *^a^1.05* | *^a^1.26* | *^a^1.14* |
| *NC178 | m, 23 | 2.36 | *mBM347 | f, 28 | 18.98 | *^b^0.95* | *^b^0.81* | *^b^0.79* | *0.88* |

*Indicates experiment where the total cell number was 0.5 x10^6^

CPD = Cumulative population doubling

^a^Responders

^b^Non-Responders
