# Supplementary material for: Suppression of Hypertrophy During in vitro Chondrogenesis of Cocultures of Human Mesenchymal Stem Cells and Nasal Chondrocytes Correlates With Lack of in vivo Calcification and Vascular Invasion
Source: Front Bioeng Biotechnol. 2021 Jan 5;8:572356. doi: 10.3389/fbioe.2020.572356 (PMC7813892; doi:10.3389/fbioe.2020.572356)
Supplement: Supplementary file 4 [file Table_4.DOCX]

**Supplementary Table S2.** Human nasal chondrocytes (NC) and bone marrow mesenchymal stem cells (BM-MSC) coculture paring information with anonymized donor information related to *in vivo* animal study

| **NC**  **donor** | **NC**  **(sex, age)** | **NC**  **CPD** | **BM-MSC**  **donor** | **BM-MSC**  **(sex, age)** | **BM-MSC**  **CPD** | **Control interaction index** | **2 ng/ml PTHrP**  **interaction**  **index** | **200 ng/ml PTHrP interaction index** |
| --- | --- | --- | --- | --- | --- | --- | --- | --- |
| *NC177 | f, 27 | 3.02 | *mBM320 | f, 26 | 16.30 | *1.50* | *2.05* | *2.19* |
| *NC196 | m, 39 | 1.62 | *mBM350 | m, 57 | 15.47 | *1.76* | *1.56* | *1.50* |
| *NC197 | m, 51 | 2.49 | *mBM320 | f, 26 | 16.30 | *2.33* | *2.62* | *2.51* |

*Indicates experiment where the total cell number was 0.5 x10^6^

CPD = Cumulative population doubling
