# Supplementary material for: Suppression of Hypertrophy During in vitro Chondrogenesis of Cocultures of Human Mesenchymal Stem Cells and Nasal Chondrocytes Correlates With Lack of in vivo Calcification and Vascular Invasion
Source: Front Bioeng Biotechnol. 2021 Jan 5;8:572356. doi: 10.3389/fbioe.2020.572356 (PMC7813892; doi:10.3389/fbioe.2020.572356)
Supplement: Supplementary file 5 [file Table_5.DOCX]

**Supplementary Table S3.** Primer sequences for quantitative real-time PCR

| **Gene** | **Accession#** | **5’ primer sequence** | **3’primer sequence** |
| --- | --- | --- | --- |
| *ACAN* | NM_001135.3 | AGGGCGAGTGGAATGATGTT | GGTGGCTGTGCCCTTTTTAC |
| *ALPL* | NM_000478.6 | CCTGGCAGGGCTCACACT | AAACAGGAGAGTCGCTTCAGAGA |
| *B2M* | NM_004048.3 | TGCTGTCTCCATGTTTGATGTATCT | TCTCTGCTCCCCACCTCTAAGT |
| *COL1A2* | NM_000089.3 | GCTACCCAACTTGCCTTCATG | GCAGTGGTAGGTGATGTTCTGAGA |
| *COL2A1* | NM_001844.5 | CTGCAAAATAAAATCTCGGTGTTCT | GGGCATTTGACTCACACCAGT |
| *COL10A1* | NM_000493.3 | GAAGTTATAATTTACACTGAGGGTTTCAAA | GAGGCACAGCTTAAAAGTTTTAAACA |
| *HMBS* | NM_000190.4 | GGCAATGCGGCTGCAA | GGGTACCCACGCGAATCAC |
| *ID1* | NM_002165.4 | ACGTGCTGCTCTACGACATGA | TGGGCACCAGCTCCTTGA |
| *IHH* | NM 002181.4 | CCTTGTCAGCCGTGAGGCCG | GCTGCCGGCTCCGTGTGATT |
| *MMP13* | NM_002427.4 | CATCCAAAAACGCCAGACAA | CGGAGACTGGTAATGGCATCA |
| *SOX9* | NM_000346.3 | CTTTGGTTTGTGTTCGTGTTTTG | AGAGAAAGAAAAAGGGAAAGGTAAGTTT |
| *YWHAZ* | NM_003406.3 | TCTGTCTTGTCACCAACCATTCTT | TCATGCGGCCTTTTTCCA |
