# Supplementary material for: Suppression of Hypertrophy During in vitro Chondrogenesis of Cocultures of Human Mesenchymal Stem Cells and Nasal Chondrocytes Correlates With Lack of in vivo Calcification and Vascular Invasion
Source: Front Bioeng Biotechnol. 2021 Jan 5;8:572356. doi: 10.3389/fbioe.2020.572356 (PMC7813892; doi:10.3389/fbioe.2020.572356)

**Supplementary Table S7**. Pearson’s correlation statistics between PTHrP concentration and measured gene expression of markers of non-hypertrophic and hypertrophic markers of chondrogenesis in cocultures of nasal chondrocytes (NC) and bone marrow mesenchymal stem cells (BM-MSC) that resulted in interaction indices < 1 (i.e. Non-Responders)


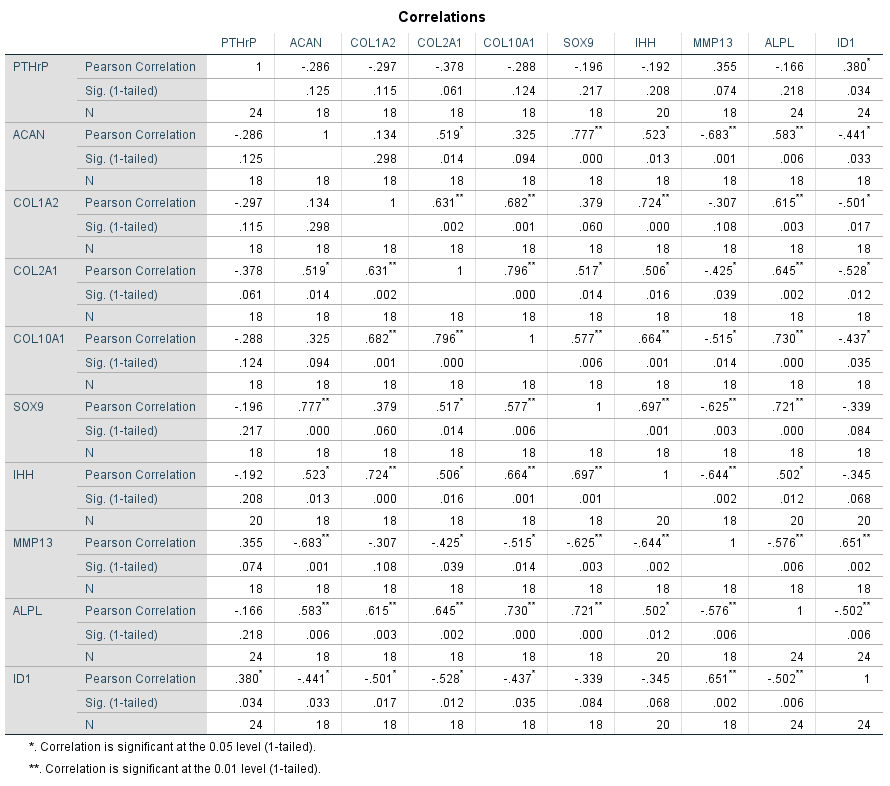

Supplement: Supplementary file 9 [file Table_9.DOCX]
