# Supplementary material for: Suppression of Hypertrophy During in vitro Chondrogenesis of Cocultures of Human Mesenchymal Stem Cells and Nasal Chondrocytes Correlates With Lack of in vivo Calcification and Vascular Invasion
Source: Front Bioeng Biotechnol. 2021 Jan 5;8:572356. doi: 10.3389/fbioe.2020.572356 (PMC7813892; doi:10.3389/fbioe.2020.572356)

**Supplementary Table S8**. Kaiser-Meyer-Olkin (KMO) measure of sampling adequacy to perform principal component analysis (PCA) on the correlations of PTHrP concentration and measured gene expression of markers of non-hypertrophic and hypertrophic markers of chondrogenesis in cocultures of nasal chondrocytes (NC) and bone marrow mesenchymal stem cells (BM-MSC) resulting in interaction indices < 1 (i.e. Non-Responders).


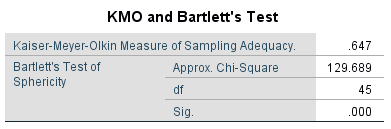

Supplement: Supplementary file 10 [file Table_10.DOCX]
